# Vitamin D deficiency in hip fracture patients is associated with an increased mortality risk

**DOI:** 10.1007/s00590-024-04162-8

**Published:** 2024-12-02

**Authors:** John M. Bayram, Hariprasath Kanesan, Nicholas D. Clement

**Affiliations:** Edinburgh Orthopaedics, Royal Infirmary of Edinburgh, Edinburgh, EH16 4SA UK

**Keywords:** Hip, Fracture, Mortality, Vitamin D Deficiency, Outcome, Falls

## Abstract

**Purpose:**

The aims were to assess whether vitamin D deficiency influenced mortality risk and length of acute hospital stay in patients presenting with a hip fracture.

**Methods:**

A retrospective study was undertaken including all patients aged over 50 years that were admitted with a hip fracture to a single centre during a 24-month period. Serum vitamin D levels on admission, patient demographics, perioperative variables and mortality were collected. Cox regression analysis was utilised to determine the independent association between serum vitamin D levels and patient mortality.

**Results:**

The cohort consisted of 1510 patients with a mean age of 81.3 years and 1107 (71.4%) were female. 876 (58.0%) were vitamin D deficient (< 50 nmol/l). The median follow up was 405 (IQR 249 to 610) days. During follow-up there were 464 deaths (30.7%). Vitamin D deficiency was independently associated with higher mortality risk (hazard ratio [HR] 1.26, 95% confidence interval (CI) 1.03 to 1.53, *P* = 0.022). Male sex (HR 1.64, 95% CI 1.34 to 2.01, *P* < 0.001) was also associated with a higher mortality risk. Vitamin D deficiency was not associated with length of hospital stay (median difference 0 days, *P* = 0.207).

**Conclusion:**

Vitamin D deficiency was independently associated with increased mortality in hip fracture patients, though this finding may be influenced by lack of comprehensive adjustment for comorbidity. While the value of routine serum vitamin D measurement is debated, supplementation during hospital stays is important to reduce falls and fracture risks associated with deficiency.

## Introduction

Vitamin D deficiency has been described as a pandemic which affects approximately 40% of Europeans, with associated costs estimated to be in excess of a billion Euros [1, 2]. Vitamin D deficiency is defined by the Endocrine Society Task Force as a serum 25-hydroxyvitamin D [25(OH)D] level < 50 nmol/l [3]. The optimal level is often described as being higher than this with 75 nmol/l as a common target [4] as at this level vitamin D is thought to help prevent fractures [5]. Vitamin D is primarily obtained through sunlight exposure, specifically to ultraviolet B radiation, but it can also obtained in lower amounts through diet and supplementation [6]. Due to its northern latitude [7], Scotland is a particular endemic risk area for vitamin D deficiency. The mean serum vitamin D level in Scotland is 37.5 nmol/l, and 75% (in summer) to 92% (in winter) of Scottish people have suboptimal vitamin D levels (< 75 nmol/l) [8, 9]. Socioeconomic deprivation was also associated with vitamin D deficiency in Scotland, with those living in the most deprived areas having the lowest vitamin D levels [8, 9].

Vitamin D is involved in a wide range of cellular functions, with the vitamin D receptors being found in nearly all human cells [10]. Its primary function is calcium homeostasis and maintenance of bone mineral density, but it also has roles in neuromuscular and cognitive function, immune system regulation and glucose metabolism [10, 11]. In addition to osteoporosis and muscular weakness [12], vitamin D deficiency has been linked to most of the leading causes of death in high income countries [13] including heart disease, stroke, cancer, diabetes [14], chronic obstructive pulmonary disease [15] and dementia [16]. It is therefore unsurprising that vitamin D deficiency is associated with all-cause mortality [17], but whether or not the link between vitamin D deficiency, chronic disease and mortality is causal or associative is unknown [4]. Vitamin D supplementation has however proven to reduce fracture and falls risks in frail elderly populations [5, 18, 19], and it is therefore now a national standard in Scotland to administer a loading dose of vitamin D to all hip fracture patients [20].

The primary aim of this study was to assess whether patients with a hip fracture who were vitamin D deficient had an increased mortality risk following their injury. The null hypothesis was that there was no difference in mortality for patients who were vitamin D deficient compared to those who were not deficient. The secondary aim was to assess whether being vitamin D deficient influenced length of acute hospital stay.

## Methods

This retrospective study included all patients aged 50 years or more that were admitted with a hip fracture to the study centre over a 24-month period (1st July 2020 to 30th June 2022). The study centre is the only trauma centre serving a catchment population of approximately 850,000 and manages over one thousand hip fractures per year. The inclusion criteria were patients with either an intracapsular or extracapsular (no more than five centimetres of distal extension from the lesser trochanter) fracture of the proximal femur, that were resident in the catchment population and had presented to the emergency department (ED). Patients with isolated fractures of the acetabulum, pubic ramus, greater trochanter and periprosthetic fractures were excluded.

Patients were retrospectively identified from the local hip fracture database which was collected prospectively on a continuous basis for submission to the Scottish Hip Fracture Audit (SHFA) and was inclusive of all patients. Patient demographics, place of domicile, fracture type, ED admission and discharge times, delirium status, time to theatre, American Society of Anaesthesiologists (ASA) grade, length of stay, mortality and serum vitamin D level on ward admission was collected from the patients e-health records (TRAKcare) and service documentation. The ASA grade was obtained from the anaesthetic notes, recorded at the time of surgery. Time to theatre was taken as per the SHFA guidelines; from time of admission to the ward to commencement of anaesthesia. Vitamin D deficiency was defined as per the Endocrine Society Task Force definition of a serum 25-hydroxyvitamin D [25(OH)D] level < 50 nmol/l [3]. These data were compiled by specialist local audit coordinators familiar with hip fractures and the trauma unit. The data were collected and assessed for completeness by a senior analyst as part of the routine activity of the SHFA. All data were handled in accordance with the UK Caldicott principles.

Throughout the study period, it was protocol to take serum vitamin D levels for all hip fracture patients. Vitamin D deficiency in these patients was managed with a combined oral calcium and vitamin D supplement. Bisphosphonates were not routinely prescribed based on vitamin D status during the study period.

ASA grade was used as a measure of each patient’s overall health and comorbidity. It is a subjective assessment made by the anaesthetist that classifies a patient’s severity of systemic disease from 1 (completely healthy and fit) to 5 (moribund and not expected to live 24 h without surgery) [21]. More detailed measures of comorbidity, including body mass index (BMI), are not routinely collected by the SHFA and therefore were not available for analysis.

The Scottish Index of Multiple Deprivation (SIMD) was used to assign the socioeconomic status of each patient with assessment of seven domains: current income, employment, health, education, skills and training, housing, geographic access and crime [22]. The current study used the updated SIMD rankings published in 2020 to assign a patient to a quintile of local data zone deprivations (1 = most deprived to 5 = least deprived) according to their postcode at time of injury.

The four “A’s” test (4AT) is used internationally as a validated clinical tool for detecting delirium [23]. A score of 4 or more is suggestive of delirium but is not diagnostic. The 4AT is assessed and recorded as part of the “standard” of care for the SHFA as a screening tool for delirium.

### Outcomes

Acute length of hospital stay (LOS) was defined as the number of days between admission to discharge from the trauma unit. Patient mortality status was obtained from the study centre hospital electronic records which is the sole national health service provider for the catchment population.

### Statistics

Statistical analysis was performed using R software (R foundation for statistical computing, Vienna, Austria) version 4.4.1. Descriptive statistics were used to describe data. Unpaired t-tests and Mann–Whitney U tests were used to assess for significant differences between groups in normally (age) and non-normally distributed (time to theatre, time in ED) continuous variables, respectively. Between group comparisons for dichotomous variables were assessed using a chi square test (sex, SIMD, ASA grade, pre-fracture residence, fracture type, 4AT score). Kaplan–Meier time to event methodology was used to assess patient survival post-operatively. Cox proportional hazard analysis was used to assess mortality risk and the independent association of factors influencing patient mortality when adjusting for confounding variables. A p-value of < 0.05 was defined as statistically significant.

## Results

**Fig. 1:**
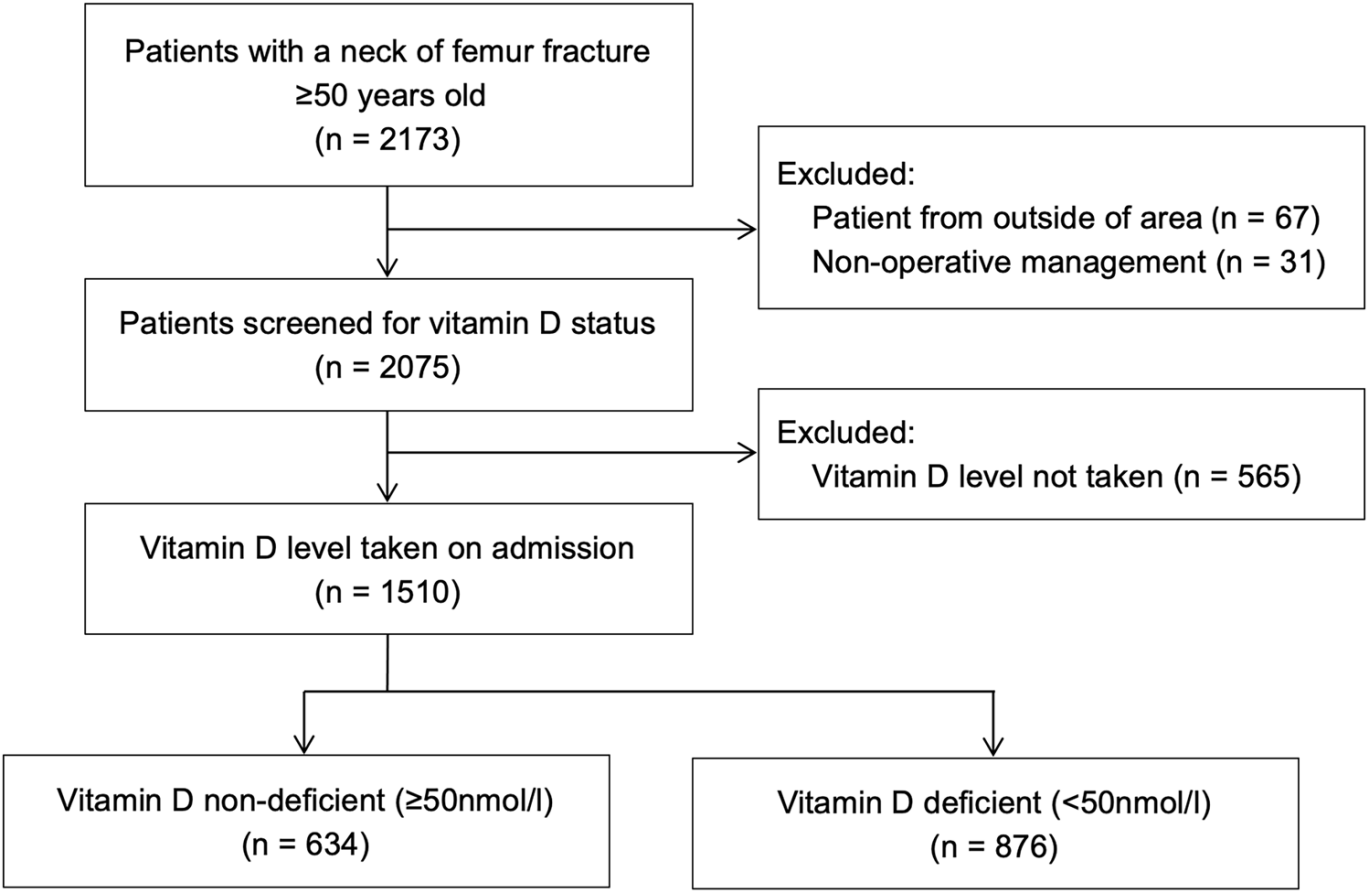
Patient inclusion flowchart

There were 2173 patients admitted with a hip fracture during the study period, of which 67 (3.1%) were from outside the catchment population of the study centre and were excluded. There were a further 31 patients (1.4%) that were managed non-operatively and excluded. Of the remaining 2075 patients, 1510 (72.8%) had a vitamin D level taken on admission and 565 (27.2%) did not (Fig. 1). The differences between these groups are shown in Table 1.

**Table 1:** Patient demographics, ASA grade, place of domicile, fracture type, delirium status according to the 4AT, and time to theatre grouped by if a Vitamin D level was taken or not

Demographic	Descriptive	Group		Difference/Odds Ratio (95% CI^a^)	p-value
		Vitamin D level taken (n = 1510)	Vitamin D level not taken (n = 565)		
Sex (n, % of group)	Female	1107 (73.3)	364 (64.4)	Reference	
	Male	403 (26.7)	201 (35.6)	1.52 (1.23 to 1.86)	< 0.001*
Age (years: mean, SD)		81.3 (9.4)	79.3 (11.5)	− 2.0 (− 0.9 to − 3.1)	< 0.001**
SIMD (n, % of group)	1 (Most)	177 (11.7)	76 (13.5)	1.17 (0.85 to 1.60)	0.344*
	2	302 (20.0)	125 (22.1)	1.12 (0.86 to 1.47)	0.393*
	3	264 (17.5)	85 (15.0)	0.87 (0.65 to 1.17)	0.374*
	4	254 (16.8)	90 (15.9)	0.96 (0.72 to 1.29)	0.797*
	5 (Least)	513 (34.0)	189 (33.5)	Reference	
ASA Grade (n, % of group)	1	27 (1.8)	21 (3.7)	Reference	
	2	387 (25.6)	134 (23.7)	0.45 (0.24 to 0.82)	0.011*
	3	956 (63.3)	347 (61.4)	0.47 (0.26 to 0.85)	0.012*
	4	129 (8.5)	61 (10.8)	0.61 (0.32 to 1.17)	0.137*
	Missing	11 (0.7)	2 (0.4)	0.25 (0.03 to 1.09)	0.105*
Pre-fracture Residence (n, % of group)	Home	1188 (78.7)	382 (67.6)	Reference	
	Care home	193 (12.8)	134 (23.7)	2.16 (1.68 to 2.77)	< 0.001*
	Hospital	80 (5.3)	32 (5.7)	1.25 (0.80 to 1.89)	0.317*
	Rehab	29 (1.9)	11 (1.9)	1.19 (0.56 to 2.35)	0.635*
	Other	20 (1.3)	6 (1.1)	0.95 (0.34 to 2.27)	0.914*
Fracture type (n, % of group)	Intracapsular	888 (58.8)	349 (61.8)	Reference	
	Extracapsular	622 (41.2)	216 (38.2)	0.88 (0.72 to 1.08)	0.241*
4AT (n, % of group)	0–3	935 (61.9)	308 (54.5)	Reference	
	4 +	233 (15.4)	124 (21.9)	1.62 (1.25 to 2.08)	< 0.001*
	Missing	342 (22.6)	133 (23.5)	1.18 (0.93 to 1.50)	0.174*
Time to Theatre (hours: median, IQR)		24.7 (16.6 to 37.3)	25.3 (17.1 to 38.8)	0.6	0.238***
Time in ED (hours: median, IQR)		4.4 (3.4 to 6.5)	4.0 (3.4 to 5.8)	−0.4	0.002***

^a^95% CI = 95% confidence interval

^*^chi-square test **Student’s t-test ^***^Mann–Whitney U test

In the study cohort of 1510 hip fracture patients with a vitamin D level taken on admission, there were 403 (28.6%) males and 1107 (71.4%) females with an overall mean age of 81.3 (SD 9.4). Of these patients, 876 (58.0%) were vitamin D deficient (< 50 nmol/l) and 634 (42.0%) were not deficient (≥ 50 nmol/l). The deficient group had a greater proportion of male patients (30.8%) compared to the non-deficient group (21.0%, *P* < 0.001). There was also a greater proportion of intracapsular fractures (61.3%) in the deficient group compared to the non-deficient group (55.4%, *P* = 0.024). Delirium, defined by a 4AT score of 4 or more, was more common in the deficient group (17.0%) compared to the non-deficient group (13.2%). The deficient group also spent longer in the ED (median = 4.9 h, interquartile range (IQR) 3.5 to 7.2) compared to the non-deficient group (median = 4.0 h, IQR 3.3 to 5.7; *P* < 0.001). There were no significant differences in age, SIMD, ASA grade, pre-fracture residence, or time-to-theatre between deficient and non-deficient groups (Table 2).

**Table 2:** Patient demographics, ASA grade, place of domicile, fracture type, delirium status according to the 4AT, and time to theatre grouped by vitamin D status

Demographic	Descriptive	Group		Difference/Odds Ratio (95% CI^a^)	p-value
		Vitamin D Not deficient (n = 634)	Vitamin D Deficient (n = 876)		
Sex (n, % of group)	Female	501 (79.0)	606 (69.2)	Reference	
	Male	133 (21.0)	270 (30.8)	1.68 (1.32 to 2.13)	< 0.001*
Age (years: mean, SD)		81.3 (9.1)	81.3 (9.7)	0.0 (−1.0 to 0.9)	0.948**
SIMD (n, % of group)	1 (Most)	72 (11.4)	105 (12.0)	1.18 (0.84 to 1.68)	0.338*
	2	118 (18.6)	184 (21.0)	1.27 (0.95 to 1.69)	0.109*
	3	105 (16.6)	159 (18.2)	1.23 (0.91 to 1.67)	0.178*
	4	109 (17.2)	145 (16.6)	1.08 (0.80 to 1.47)	0.616*
	5 (Least)	230 (36.3)	283 (32.3)	Reference	
ASA Grade (n, % of group)	1	12 (1.9)	15 (1.7)	Reference	
	2	180 (28.4)	207 (23.6)	0.922 (0.41 to 2.03)	0.842*
	3	385 (60.7)	571 (65.2)	1.19 (0.54 to 2.58)	0.663*
	4	51 (8.0)	78 (8.9)	1.22 (0.52 to 2.85)	0.640*
	Missing	6 (0.9)	5 (0.6)	0.68 (0.15 to 2.87)	0.595*
Pre-fracture Residence (n, % of group)	Home	511 (80.6)	677 (77.3)	Reference	
	Care home	71 (11.2)	122 (13.9)	1.30 (0.95 to 1.78)	0.104*
	Hospital	28 (4.4)	52 (5.9)	1.40 (0.88 to 2.28)	0.162*
	Rehab	15 (2.4)	14 (1.6)	0.71 (0.33 to 1.49)	0.358*
	Other	9 (1.4)	11 (1.3)	0.92 (0.37 to 2.33)	0.855*
Fracture type (n, % of group)	Intracapsular	351 (55.4)	537 (61.3)	Reference	
	Extracapsular	283 (44.6)	339 (38.7)	0.78 (0.64 to 0.96)	0.024*
4AT (n, % of group)	0–3	410 (64.7)	525 (59.9)	Reference	
	4 +	84 (13.2)	149 (17.0)	1.38 (1.03 to 1.87)	0.031*
	Missing	140 (22.1)	202 (23.1)	1.12 (0.88 to 1.45)	0.353*
Time to Theatre (hours: median, IQR)		25.4 (17.6 to 38.1)	23.6 (16.3 to 36.5)	−1.8	0.015***
Time in ED (hours: median, IQR)		4.0 (3.3 to 5.7)	4.9 (3.5 to 7.2)	0.9	< 0.001***

^a^95% CI = 95% confidence interval

^*^chi square test **Student’s t-test ^***^Mann–Whitney U test

**Fig. 2:**
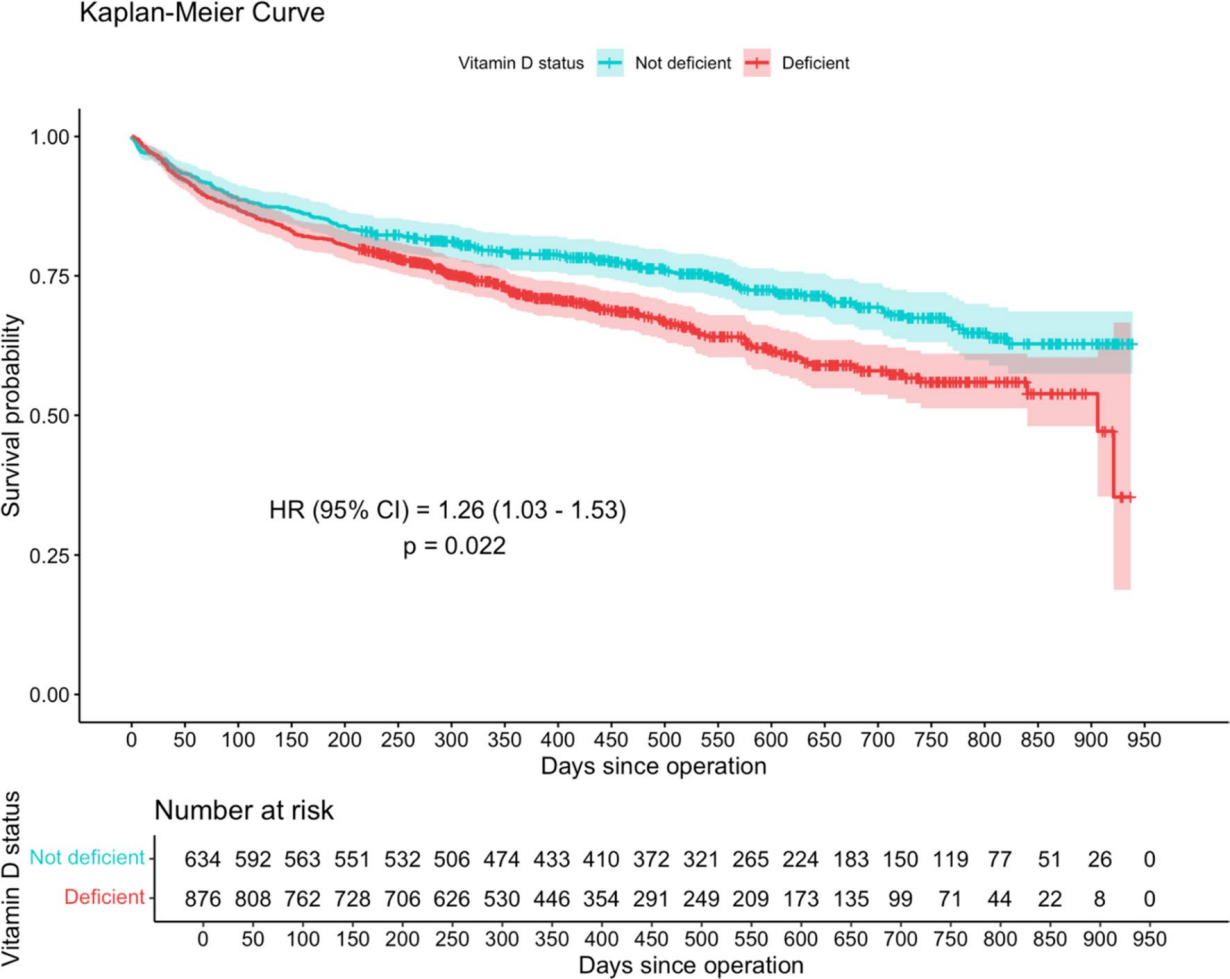
Kaplan–Meier survival curve for hip fracture patients grouped by vitamin D status. The numbers of patients at risk at 50-day intervals since the day of surgery are shown in the table

**Fig. 3:**
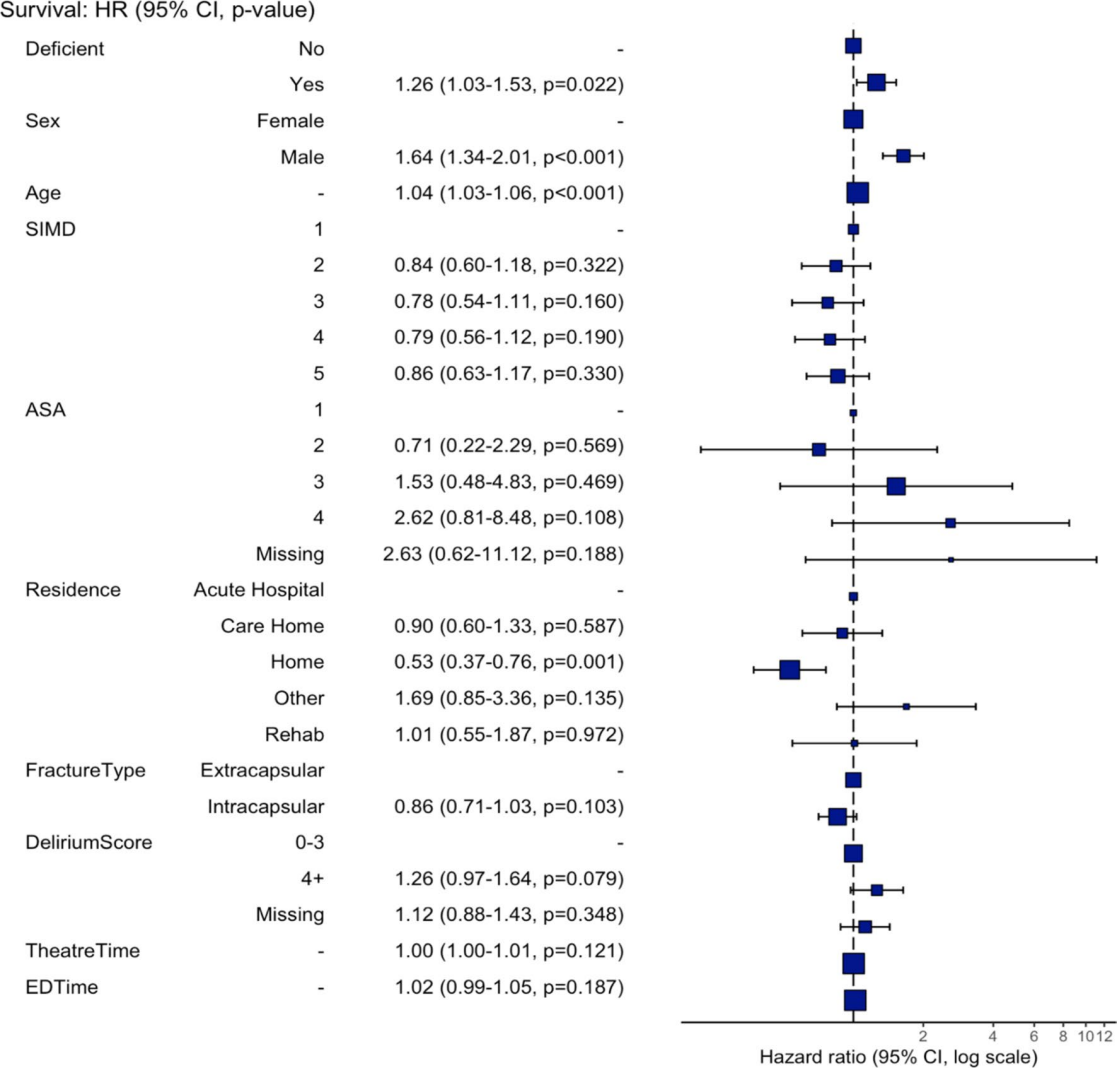
Hazard ratios from cox regression model for variables associated with patient mortality over the follow-up period following a hip fracture. HR = hazard ratio. 95% CI = 95% confidence interval

The median follow up was 405 (IQR 249 to 610) days, and during the follow up period there were 464 deaths (30.7%). Cox proportional hazard analysis showed that vitamin D deficient patients had a 1.26 times higher risk of death over the follow-up period compared to non-deficient patients (hazard ratio [HR] 1.26, 95% confidence interval (CI) 1.03 to 1.53, *P* = 0.022; Fig. 2). Male sex (HR 1.64, 95% CI 1.34 to 2.01, *P* < 0.001) was also associated with a higher risk of death (Fig. 3). Conversely, living at home prior to fracture was protective against death (HR 0.53, 95% CI 0.37 to 0.76, *P* = 0.001). Survival at 1 year was 71.1% (95% CI 68.6 to 74.9) for vitamin D deficient patients whereas for those who were not deficient survival was 79.0% (95% CI 75.9 to 82.3; Table 3). The survival difference between groups widens even further at 2 years with vitamin D deficient patients at 56.7% survival (95% CI 52.2 to 61.6) compared to non-deficient patients at 67.4% survival (95% CI 63.1 to 72.1).

**Table 3:** Patient survival following a hip fracture at different timepoints according to group

Timepoint	Group Survival (%, 95% CI^a^)	
	Vitamin D not deficient	Vitamin D deficient
30-day	95.9 (94.4 to 97.5)	95.2 (93.8 to 96.6)
60-day	92.6 (90.6 to 94.6)	90.5 (88.6 to 92.5)
90-day	89.6 (87.2 to 92.0)	87.7 (85.5 to 89.9)
1-year	79.0 (75.9 to 82.3)	71.7 (68.6 to 74.9)
2-year	67.4 (63.1 to 72.1)	56.7 (52.2 to 61.6)
Final follow-up	62.8 (57.5 to 68.6)	35.4 (18.8 to 66.6)

^a^95% CI = 95% confidence interval

Median LOS was 12 (IQR 8 to 18) days. Vitamin D deficient patients did not have any difference in LOS compared to non-deficient patients (median 12 (IQR 8 to 18) versus 12 (IQR 7 to 18) days, *P* = 0.207).

## Discussion

This study has demonstrated that patients with a hip fracture who are vitamin D deficient had a higher mortality risk over the follow-up period, independent of confounding variables. 1-year and 2-year survival for vitamin D deficient patients were worse than non-deficient patients by 7.9% and 10.7% respectively. The vitamin D deficient group in this study had a greater proportion of male patients (30.8% vs 21.0%) and a higher incidence of delirium (17.0% vs 13.2%). This is in keeping with previous studies [24] and suggests that this group may be frailer than the non-deficient group [25, 26], a known predictor of mortality in hip fracture patients [27]. The vitamin D deficient group also spent longer in the ED (4.9 h vs 4 h), had a greater proportion of intracapsular fractures (61.3% vs. 55.4%), and experienced a shorter wait time for surgery (23.6 vs 25.4 h). In contrast, previous studies have found no association between vitamin D status and fracture pattern [24]. These results may be due to type 1 error, highlighting the need for further research in this area. Male patients in this study had a 1.64 times higher risk of death, which is in keeping with previous studies showing male sex as a risk factor for mortality in this population [28, 29]. Vitamin D deficiency was not associated with any difference in length of hospital stay.

Our findings are broadly consistent with previous published work examining the impact of vitamin D deficiency on mortality and length of hospital stay in hip fracture patients. A recent review and meta-analysis of nine studies and 4409 patients showed that vitamin D insufficient (50–75 nmol/l) and severely deficient (< 25 nmol/l) hip fracture patients had an increased mortality risk, however these differences were no longer present when adjusting for the confounding variables of age, sex, serum albumin levels and co-morbidities [30]. In keeping with our findings, another recent meta-analysis of seven studies (1972 patients) showed that vitamin D deficiency had no impact on length of hospital stay [31].

There are multiple possible explanations for the variability in the findings of this study and those within the literature. Firstly, chronic diseases are a major predictor of post-operative mortality in the hip fracture population [32]. Vitamin D deficiency is correlated with many chronic diseases including heart disease, stroke, cancer, diabetes [14], chronic obstructive pulmonary disease [15] and dementia [16]. Adjusting for the mortality risk of chronic diseases is a challenge, and few studies have used a comprehensive measure such as the Charlson Comorbidity Index to adjust for confounding [33–35]. Although ASA grade of 3 or greater is a strong predictor of mortality in this population [32], it seems possible that this measure in isolation may not be able to fully adjust for the mortality risk of all relevant chronic diseases.

The increased mortality in vitamin D deficient patients with a hip fracture could also be related to the associations between vitamin D deficiency, increased clinical frailty [36], reduced functional ability [31], and increased falls and fracture risks [5, 18, 37]. Increased clinical frailty, of which functional ability is a major component [38], is highly predictive of post-operative mortality in hip fracture patients [39]. Subsequent fall and re-fracture following hip fracture surgery is also unsurprisingly associated with increased mortality [40]. Differentiating association from causation with these interrelated risk factors is challenging. Vitamin D appears to have a vital role in the maintenance of strength, function, balance and bone mineral density in elderly patients, as vitamin D supplementation has been shown to reduce falls and fracture risks [5, 18]. However, combined oral calcium and vitamin D supplementation following hip fracture in this study did not result in equivalent mortality between deficient and non-deficient patients. This suggests that the increased mortality risk associated with vitamin D deficiency is not completely reversible with oral supplementation alone. Current SHFA guidelines now recommend the routine administration of an intravenous bisphosphonate for all hip fracture patients in addition to oral calcium and vitamin D supplementation [20]. This may improve the mortality rates associated with vitamin D deficiency observed in this study due to both the skeletal and non-skeletal benefits of bisphosphonate therapy [41].

This study was limited by its retrospective single centre design. The confounders used in this study may also have not completely accounted for the mortality risks associated with chronic diseases that vitamin D deficiency is associated with. This study was also prone to selection bias, as 72.8% of hip fracture patients over the study period had a vitamin D level taken on admission. Patients with vitamin D levels taken were slightly older, had a lower likelihood of being ASA grade 1, were less likely to be admitted from a care home, had a lower incidence of delirium, and spent more time in ED. This may indicate a selection bias influenced by admitting doctors' perceptions of the relevance of measuring vitamin D levels, potentially based on the patient's age, fitness, or a need to prioritise other investigations, such as those for delirium. Additionally, a single serum measurement of vitamin D level at the time of an acute injury is an imperfect surrogate of vitamin D status. High C-reactive protein and low albumin levels can lower serum vitamin D levels [42], and therefore concurrent acute illnesses may have influenced results in some patients.

In conclusion, there was an independent association between vitamin D deficiency and increased mortality in hip fracture patients. These results agree with the wider literature, but the independence from confounders may be due to lack of inclusion of a comprehensive measure of comorbidity in this study. The role of routinely measuring vitamin D status in this population is debated. While some consider it mandatory [43], others, like the SHFA [20], consider vitamin D deficiency so prevalent that measurement is unnecessary and treat all hip fracture patients for vitamin D deficiency. Ensuring hip fracture patients receive vitamin D supplementation during their hospital stay remains important given its high prevalence and the increased mortality, falls and fracture risks linked to vitamin D deficiency.

## Data Availability

No datasets were generated or analysed during the current study.

## References

[1] Cashman KD, Dowling KG, Škrabáková Z et al (2016) Vitamin D deficiency in Europe: pandemic? Am J Clin Nutr 103:1033–1044. 10.3945/ajcn.115.120873 26864360 10.3945/ajcn.115.120873 PMC5527850

[2] Grant WB, Cross HS, Garland CF et al (2009) Estimated benefit of increased vitamin D status in reducing the economic burden of disease in western Europe. Prog Biophys Mol Biol 99:104–113. 10.1016/j.pbiomolbio.2009.02.003 19268496 10.1016/j.pbiomolbio.2009.02.003

[3] Holick MF, Binkley NC, Bischoff-Ferrari HA et al (2011) Evaluation, treatment, and prevention of vitamin d deficiency: an endocrine society clinical practice guideline. J Clin Endocrinol Metab 96:1911–1930. 10.1210/jc.2011-0385 21646368 10.1210/jc.2011-0385

[4] Amrein K, Scherkl M, Hoffmann M et al (2020) Vitamin D deficiency 2.0: an update on the current status worldwide. Eur J Clin Nutr 74:1498–1513. 10.1038/s41430-020-0558-y 31959942 10.1038/s41430-020-0558-y PMC7091696

[5] Bischoff-Ferrari HA, Willett WC, Wong JB et al (2005) Fracture prevention with vitamin D supplementation A meta-analysis of randomized controlled trials. JAMA 293:2257–2264. 10.1001/jama.293.18.2257 15886381 10.1001/jama.293.18.2257

[6] Benedik E (2022) Sources of vitamin D for humans. Int J Vitam Nutr Res 92:118–125. 10.1024/0300-9831/a000733 34658250 10.1024/0300-9831/a000733

[7] Huotari A, Herzig K-H (2008) Vitamin D and living in northern latitudes—an endemic risk area for vitamin D deficiency. Int J Circumpolar Health 67:164–178. 10.3402/ijch.v67i2-3.18258 18767337 10.3402/ijch.v67i2-3.18258

[8] The Scottish Public Health Observatory (2023) Vitamin D: data. www.scotpho.org.uk/archive/vitamin-d/data/. Accessed 3 Sep 2024

[9] Purdon G, Comrie F, Rutherford L, Marcinkiewicz A (2013) Vitamin D status of Scottish adults: Results from the 2010 & 2011 Scottish Health Surveys. Food Standards Agency in Scotland, ScotCen Social Research

[10] Bikle DD (2020) Vitamin D: newer concepts of its metabolism and function at the basic and clinical level. J Endocrine Soc 4:bvz038. 10.1210/jendso/bvz038 32051922 10.1210/jendso/bvz038 PMC7007804

[11] Mitri J, Dawson-Hughes B, Hu FB, Pittas AG (2011) Effects of vitamin D and calcium supplementation on pancreatic β cell function, insulin sensitivity, and glycemia in adults at high risk of diabetes: the Calcium and Vitamin D for Diabetes Mellitus (CaDDM) randomized controlled trial1234. Am J Clin Nutr 94:486–494. 10.3945/ajcn.111.011684 21715514 10.3945/ajcn.111.011684 PMC3142723

[12] Sanders KM, Scott D, Ebeling PR (2014) Vitamin D deficiency and its role in muscle-bone interactions in the elderly. Curr Osteoporos Rep 12:74–81. 10.1007/s11914-014-0193-4 24488588 10.1007/s11914-014-0193-4

[13] World Health Organization (2024) The top 10 causes of death. World Health Organization

[14] Muscogiuri G (2018) Vitamin D: past, present and future perspectives in the prevention of chronic diseases. Eur J Clin Nutr 72:1221–1225. 10.1038/s41430-018-0261-4 30185855 10.1038/s41430-018-0261-4

[15] Herr C, Greulich T, Koczulla RA et al (2011) The role of vitamin D in pulmonary disease: COPD, asthma, infection, and cancer. Respir Res 12:31. 10.1186/1465-9921-12-31 21418564 10.1186/1465-9921-12-31 PMC3071319

[16] Sultan S, Taimuri U, Basnan SA et al (2020) Low vitamin D and its association with cognitive impairment and dementia. J Aging Res 2020:6097820. 10.1155/2020/6097820 32399297 10.1155/2020/6097820 PMC7210535

[17] Pludowski P, Holick MF, Pilz S et al (2013) Vitamin D effects on musculoskeletal health, immunity, autoimmunity, cardiovascular disease, cancer, fertility, pregnancy, dementia and mortality—a review of recent evidence. Autoimmun Rev 12:976–989. 10.1016/j.autrev.2013.02.004 23542507 10.1016/j.autrev.2013.02.004

[18] Bischoff-Ferrari HA, Dawson-Hughes B, Staehelin HB et al (2009) Fall prevention with supplemental and active forms of vitamin D: a meta-analysis of randomised controlled trials. BMJ 339:b3692. 10.1136/bmj.b3692 19797342 10.1136/bmj.b3692 PMC2755728

[19] Cameron ID, Gillespie LD, Robertson MC, et al (2012) Interventions for preventing falls in older people in care facilities and hospitals. Cochrane Database Syst Rev 12:CD005465. 10.1002/14651858.CD005465.pub3 10.1002/14651858.CD005465.pub3 23235623

[20] Public Health Scotland (2024) Scottish standards of care for hip fracture patients. https://www.publichealthscotland.scot/media/27068/scottish-standards-of-care-for-hip-fracture-patients-english-may2024.pdf

[21] Dripps R (1963) New classification of physical status. Anesthesiology 24:111

[22] The Scottish Government (2020) Scottish Index of Multiple Deprivation 2020. https://www.gov.scot/collections/scottish-index-of-multiple-deprivation-2020/. Accessed 3 Sep 2024

[23] Tieges Z, Maclullich AMJ, Anand A et al (2021) Diagnostic accuracy of the 4AT for delirium detection in older adults: systematic review and meta-analysis. Age Ageing 50:733–743. 10.1093/ageing/afaa224 33951145 10.1093/ageing/afaa224 PMC8099016

[24] Ingstad F, Solberg LB, Nordsletten L et al (2021) Vitamin D status and complications, readmissions, and mortality after hip fracture. Osteoporos Int 32:873–881. 10.1007/s00198-020-05739-9 33201249 10.1007/s00198-020-05739-9

[25] Endo Y, Aharonoff GB, Zuckerman JD et al (2005) Gender differences in patients with hip fracture: a greater risk of morbidity and mortality in men. J Orthop Trauma 19:29 15668581 10.1097/00005131-200501000-00006

[26] Qi Y, Li Y, Zou J et al (2022) Risk factors for postoperative delirium in geriatric patients with hip fracture: a systematic review and meta-analysis. Front Aging Neurosci 14:523. 10.3389/fnagi.2022.960364 10.3389/fnagi.2022.960364 PMC9382199 35992597

[27] Narula S, Lawless A, D’Alessandro P et al (2020) Clinical Frailty Scale is a good predictor of mortality after proximal femur fracture: A cohort study of 30-day and one-year mortality. Bone Joint Open 1:443–449. 10.1302/2633-1462.18.BJO-2020-0089.R1 33215137 10.1302/2633-1462.18.BJO-2020-0089.R1 PMC7667224

[28] Mariconda M, Costa GG, Cerbasi S, et al (2015) The determinants of mortality and morbidity during the year following fracture of the hip: a prospective study. The Bone Joint J 97-B:383–390. 10.1302/0301-620X.97B3.34504 10.1302/0301-620X.97B3.34504 25737523

[29] Kopp L, Edelmann K, Obruba P et al (2009) Mortality risk factors in the elderly with proximal femoral fracture treated surgically. Acta Chir Orthop Traumatol Cech 76:41–46 19268048

[30] Llombart R, Mariscal G, Barrios C et al (2024) Impact of vitamin D deficiency on mortality in patients with hip fracture: a meta-analysis. J Am Geriatr Soc 72:268–279. 10.1111/jgs.18601 37772615 10.1111/jgs.18601

[31] Llombart R, Mariscal G, Barrios C et al (2024) Does vitamin D deficiency affect functional outcomes in hip fracture patients? A meta-analysis of cohort studies. J Endocrinol Invest 47:1323–1334. 10.1007/s40618-023-02266-2 38112912 10.1007/s40618-023-02266-2

[32] Bui M, Nijmeijer WS, Hegeman JH et al (2024) Systematic review and meta-analysis of preoperative predictors for early mortality following hip fracture surgery. Osteoporos Int 35:561–574. 10.1007/s00198-023-06942-0 37996546 10.1007/s00198-023-06942-0 PMC10957669

[33] Cher EWL, Allen JC, Moo IH et al (2020) Sub-optimal serum 25-hydroxyvitamin D level affects 2-year survival after hip fracture surgery. J Bone Miner Metab 38:555–562. 10.1007/s00774-019-01082-0 31974676 10.1007/s00774-019-01082-0

[34] Dauny V, Thietart S, Cohen-Bittan J et al (2022) Association between Vitamin D deficiency and prognosis after hip fracture surgery in older patients in a dedicated orthogeriatric care pathway. J Nutr Health Aging 26:324–331. 10.1007/s12603-022-1762-335450987 10.1007/s12603-022-1762-3

[35] Fu G, Wu R, Zhang R et al (2023) Preoperative vitamin D deficiency is associated with increased one-year mortality in chinese geriatric hip fracture patients – a propensity score matching study. Clin Interv Aging 18:263–272. 10.2147/CIA.S395228 36843634 10.2147/CIA.S395228 PMC9945644

[36] Zheng Z, Xu W, Wang F et al (2022) Association between vitamin D3 levels and frailty in the elderly: a large sample cross-sectional study. Front Nutr 9:52. 10.3389/fnut.2022.980908 10.3389/fnut.2022.980908 PMC9553132 36238456

[37] LeBoff MS, Hawkes WG, Glowacki J et al (2008) Vitamin D-deficiency and post-fracture changes in lower extremity function and falls in women with hip fractures. Osteoporos Int 19:1283–1290. 10.1007/s00198-008-0582-6 18373057 10.1007/s00198-008-0582-6 PMC2577562

[38] Rockwood K, Song X, MacKnight C et al (2005) A global clinical measure of fitness and frailty in elderly people. CMAJ 173:489–495. 10.1503/cmaj.050051 16129869 10.1503/cmaj.050051 PMC1188185

[39] Song Y, Wu Z, Huo H, Zhao P (2022) The impact of frailty on adverse outcomes in geriatric hip fracture patients: a systematic review and meta-analysis. Front Public Health 10:5. 10.3389/fpubh.2022.890652 10.3389/fpubh.2022.890652 PMC9280195 35844855

[40] Trevisan C, Bedogni M, Pavan S et al (2020) The impact of second hip fracture on rehospitalization and mortality in older adults. Arch Gerontol Geriatr 90:104175. 10.1016/j.archger.2020.104175 32659601 10.1016/j.archger.2020.104175

[41] Billington EO, Reid IR (2020) Benefits of bisphosphonate therapy: beyond the Skeleton. Curr Osteoporos Rep 18:587–596. 10.1007/s11914-020-00612-4 32734512 10.1007/s11914-020-00612-4

[42] Ghashut RA, Talwar D, Kinsella J et al (2014) The effect of the systemic inflammatory response on plasma vitamin 25 (OH) D concentrations adjusted for albumin. PLoS ONE 9:e92614. 10.1371/journal.pone.0092614 24667823 10.1371/journal.pone.0092614 PMC3965436

[43] Hershkovitz A, Maydan G, Ben Joseph R, Nissan R (2022) Vitamin D levels in post-acute hip fractured patients and their association with rehabilitation outcomes. Disabil Rehabil 44:6722–6729. 10.1080/09638288.2021.1971304 34543157 10.1080/09638288.2021.1971304

